# Evaluation of Ex-PRESS implantation combined with phacoemulsification in primary angle-closure glaucoma

**DOI:** 10.1097/MD.0000000000004613

**Published:** 2016-09-09

**Authors:** Bing Liu, Da-Dong Guo, Xiu-Juan Du, Chen-Yang Cong, Xiao-Hua Ma

**Affiliations:** a Affiliated Eye Hospital of Shandong University of Traditional Chinese Medicine; b Eye Institute of Shandong University of Traditional Chinese Medicine; c Shandong Provincial Hospital affiliated to Shandong University, Jinan, China.

**Keywords:** cataract, Ex-PRESS implantation, intraocular pressure, primary angle-closure glaucoma

## Abstract

To evaluate the safety and efficacy of Ex-PRESS (R50) implantation combined with phacoemulsification in primary angle-closure glaucoma (PACG) patients with cataract.

Twenty-four eyes of 24 patients with unregulated PACG underwent combined cataract and glaucoma surgery. After phacoemulsification and intraocular lens implantation, the Ex-PRESS (R-50) was inserted into the anterior chamber under a scleral flap. The intraocular pressure (IOP), best corrected visual acuity (BCVA), number of medications, and complications were recorded preoperatively as well as postoperatively on day 7 and at 1, 3, 6, and 12 months.

The mean follow-up was 16.4 ± 2.5 months (range 14–21 months) and the mean age of the patients was 64.7 ± 6.8 years (range 56–78 years). The mean IOP was 20.4 ± 5.4 mm Hg preoperatively and decreased to 10.2 ± 2.8, 13.1 ± 2.7, 14.9 ± 4.1, 14.3 ± 3.9, and 14.0 ± 3.6 mm Hg on day 7 and at 1, 3, 6, and 12 months after surgery (all *P* < 0.005). At 12 months, the mean BCVA was 0.62 ± 0.33 and the number of medications was 0.3 ± 0.6. Most of complications were resolved spontaneously and conservatively.

The Ex-PRESS implantation combined with phacoemulsification cataract extraction is safe and effective for reducing IOP and antiglaucoma medications in PACG patients with cataract.

## 1 Introduction

Glaucoma is the second leading cause of blindness worldwide,^[1]^ and the treatment focuses on reducing intraocular pressure (IOP), either pharmacologically or surgically. When the medication or laser treatment cannot control IOP, surgery is performed. Currently trabeculectomy is the most commonly performed glaucoma surgery and is the standard glaucoma filtration procedure.^[2]^ Its success rate and complications have been well established.^[3,4]^

In recent years, Ex-PRESS has been introduced as an alternative to trabeculectomy in reducing IOP.^[5]^ The implant is a nonvalved device that is implanted at the limbus to drain aqueous humor from the anterior chamber to the subconjunctival space, creating a conjunctival filtration bleb, which is similar to trabeculectomy.^[6]^ The device needs room in the anterior chamber angle and therefore is contraindicated in acute- or chronic-angle closure glaucoma unless concomitant cataract surgery is planned. The Ex-PRESS implant is effective in reducing the IOP either implanted alone, or in conjunction with cataract surgery.^[7]^ Over the last decade, it has been used successfully in approximately 60,000 implantations worldwide,^[8]^ and numerous studies have reported on the biocompatibility, safety, and efficacy of Ex-PRESS during its evolution.^[6,9–11]^ To the best of our knowledge, there are few survey data to evaluate the Ex-PRESS implantation combined with phacoemulsification in primary angle-closure glaucoma (PACG) patients with cataract. Therefore, the present study aimed to assess the clinical outcomes of the Ex-PRESS miniature glaucoma drainage implant placed under a scleral flap combined with phacoemulsification in PACG.

## 2 Methods

We performed a retrospective chart review of consecutive PACG patients who underwent combined Ex-PRESS implantation and cataract surgery between July 2013 and February 2014. The study protocol was approved by the Ethical Committee of Shandong University of Traditional Chinese Medicine and was performed in accordance with the tenets of the Declaration of Helsinki.

Inclusion criteria included patients with PACG and coexisting cataract. PACG was defined as the presence of glaucomatous optic neuropathy in conjunction with visual field defect, together with an occludable drainage angle (trabecular meshwork invisible for more than 270°) and other evidence of trabecular obstruction (e.g., peripheral anterior synechiae [PAS] and an IOP ≥ 21 mm Hg). Both acute and chronic PACG were defined as Sun described.^[12]^ The clinical diagnostic criteria for cataract were as follows: presence of nucleus sclerosis, cortical cataract, or subcapsular cataract confirmed by slit-lamp examination; visual acuity of 35/50 or worse. In the present study, we excluded eyes with angle closures that were secondary to other ocular abnormalities, previous incisional ocular surgery, concurrent retinal or optic neuropathy other than glaucoma, and patients with a postoperative follow-up period of <12 months.

A comprehensive examination was performed before surgery and at 7 days as well as 1, 3, 6, and 12 months after surgery. Examinations included Snellen distance best corrected visual acuity (BCVA), IOP measurement with Goldmann applanation tonometer, complications, fundus, and vertical cup/disk ratio observation. Gonioscopy, ultrasound biomicroscopy, and visual field using the Octopus 1-2-3 were performed only once before surgery.

Surgery was considered a complete success when the IOP was <21 mm Hg without medication. Qualified success was defined as IOP < 21 mm Hg with medication. Surgery was considered a failure when IOP was higher than 21 mm Hg despite medication and/or further glaucoma surgery was required or the implant was explanted. Postoperative bleb needling and fibrosis modulation with needling or 5-fluouracil (5-FU) injection (5 mg in 0.1 mL) were not a criterion for failure.

A 6-0 prolene traction suture was placed in the superior rectus muscle. After a limbus-based opening of the conjunctiva and a dissection of a 4 × 4 mm superficial scleral flap, superior sutureless clear corneal phacoemulsification cataract extraction with foldable IOL implantation was performed through a 3.0 mm incision; the ophthalmic viscosurgical device (Viscoat, Alcon Ophthalmic Product Co., Ltd., Beijing, China) was left in the anterior chamber. An incision into the anterior chamber and parallel to the iris was made using a 25-G needle, the Ex-PRESS R-50 miniature glaucoma implant (Alcon Ophthalmic Product Co., Ltd.) was then inserted according to the manufacturer's directions. The scleral flap and conjunctiva were closed with 10-0 nylon sutures. The ophthalmic viscosurgical device was cleared at the end of the operation. The standard procedure was performed without complications and all implants were well positioned. Postoperatively, patients were treated with topical tobramycin and prednisolone acetate qid for 1 month and then with topical diclofenac bid for 2 months.

Data were expressed as mean ± standard deviation. Results were analyzed using a 1-way analysis of variance and Student *t* test for parametric data (IOP), Wilcoxon signed-rank test for nonparametric data (BCVA, number of medications), and Kaplan–Meier survival curves at the end of follow-up. Results were considered significant when *P* < 0.05.

## 3 Results

A total of 24 eyes from 24 patients were included in this study. Table 1 shows the patients’ data. The mean follow-up was 16.4 ± 2.5 months (range 14–21 months). All patients completed at least 1-year follow-up.

**Table 1:**
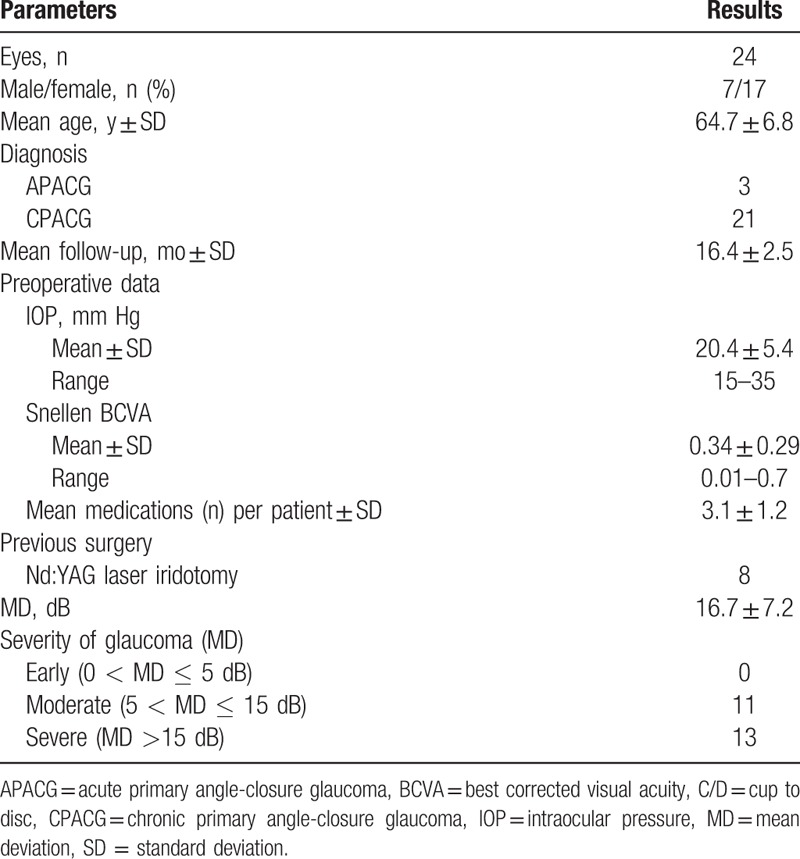
Preoperative characteristics of patients who underwent combined surgery.

**Figure 1:**
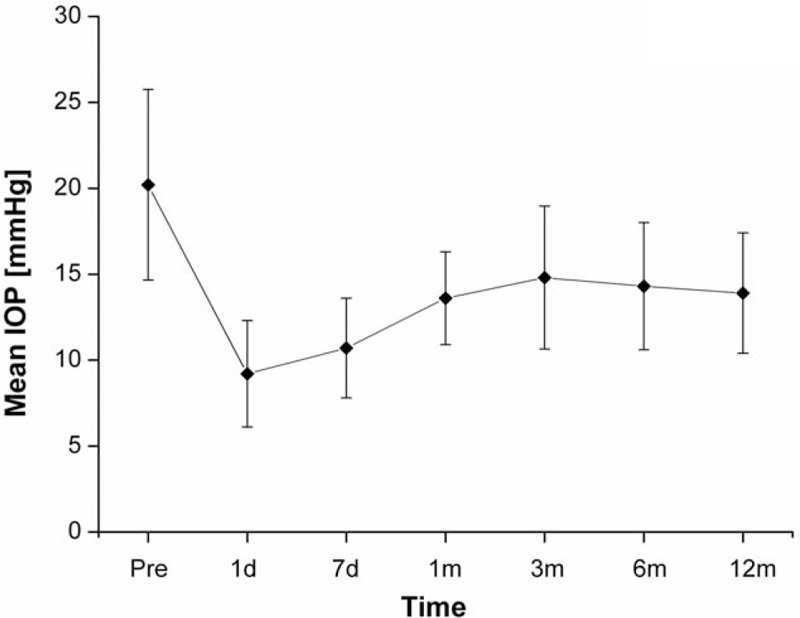
Mean IOP before surgery and at each follow-up period. The bars represent the standard deviation. d = day, IOP = intraocular pressure, m = month, Pre = preoperative IOP.

After surgery, the mean IOP was 10.2 ± 2.8 mm Hg at 7 days (*P* = 0.001), 13.1 ± 2.7 mm Hg at 1 month (*P* = 0.001), 14.9 ± 4.1 mm Hg at 3 months (*P* = 0.003), 14.3 ± 3.9 mm Hg at 6 months (*P* = 0.003), and 14.0 ± 3.6 mm Hg at 12 months (*P* = 0.001), respectively (Fig. 1). All postoperative IOP was significantly lower compared with preoperative IOP at all time points (*P* < 0.005, ANOVA and Student *t* test).

The mean preoperative BCVA (0.34 ± 0.29, range 0.01–0.7) increased to 0.62 ± 0.33 (range 0.05–1.00) at 12 months (*P* = 0.001). Visual acuity improved in 19 eyes; however, no change was recorded in 4 eyes. One patient had a BCVA dropped (worse than 0.1) as a result of choroidal detachment.

**Figure 2:**
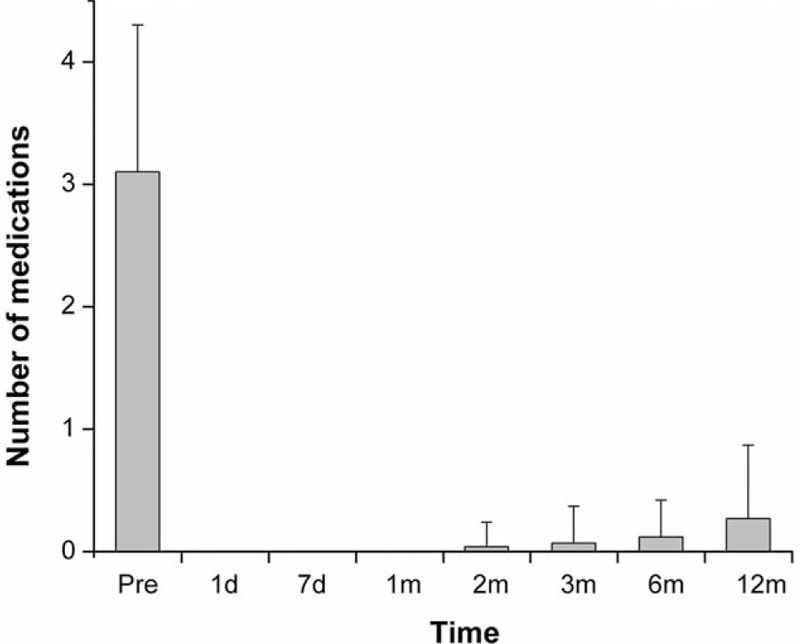
Mean number of glaucoma medications before surgery and at each follow-up period. The bars represent the standard deviation. d = day, m = month, Pre = preoperative number of antiglaucoma medications.

**Figure 3:**
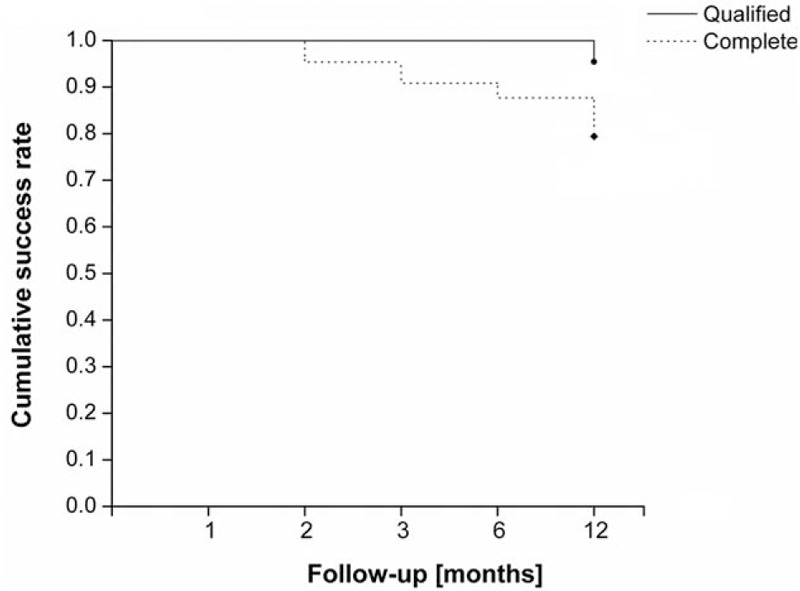
Kaplan–Meier survival analysis.

There was a significant decrease in the number of medications required after surgery (Fig. 2). During the postoperative period, there was a slight increase in number of medications required over time to achieve satisfactory IOP control. Compared to the baseline, the mean number of medications was 0.1 ± 0.3 at 6 months, but increased to 0.3 ± 0.6 at 12 months (*P* < 0.05, Wilcoxon signed-rank test). The qualified success rate was 95.8% (23/24 eyes), and the complete success rate was 79.2% at 12 months (19/24 eyes), respectively (Fig. 3).

Table 2 shows the postoperative complications. Three cases (12.5%) of flat anterior chamber were managed conservatively. Hypotony was observed in 2 eyes (8.3%) during the 1st week after the surgery without need for surgical intervention. One case (4.2%) of choroidal detachment had resolved at 6 weeks postoperatively. Of all patients, 1 case (4.2%) developed a bleb leak that required a bleb revision. Three cases (12.5%) developed posterior capsular opacities following cataract surgery, and Nd:YAG laser capsulotomy was performed to restore visual acuity. Bleb needling combined with 5-FU injection in the postoperative period was performed in 5 eyes.

**Table 2:**
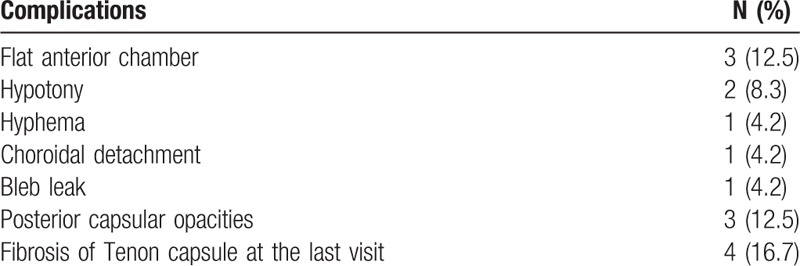
Postoperative complications after combined surgery.

## 4 Discussion

It has been accepted that PACG is higher in Asians (especially in East Asians and South East Asians) than in Europeans and Africans, with over 80% of PACG in Asia.^[13]^ In China, the prevalence of PACG was approximately double that of primary open angle glaucoma (POAG) in adults, and the increase of prevalence with age is also more prominent in PACG than POAG.^[14]^ Because PACG appears to cause blindness more frequently than POAG,^[1,15]^ thus, the treatment of PACG has been an important public health issue, and combined surgery is one commonly performed surgical option for those with coexisting cataract and medically uncontrolled primary glaucoma, with the advantage of improving visual acuity and achieving greater IOP reduction after a single surgery.^[16–19]^

In this retrospective study, a total of 24 patients (24 eyes) diagnosed with PACG were included. There were only 8 patients who had received laser iridotomy, which should be the initial treatment for PACG before they were included. For the rest of patients, preoperative examinations indicated that IOP and the progression of visual field damage could not be controlled by laser iridotomy, so combined surgery was performed. In this study, we observed successful control of IOP in the majority of patients after Ex-PRESS implantation combined with phacoemulsification, accompanied by low complication rates. Previous studies showed that Ex-PRESS shunt and standard trabeculectomy have similar efficacy and safety profiles.^[20–22]^ In our study, the reduction of IOP was significant between the preoperative and all postoperative periods. The IOP curve dropped 1 week after operation, further a slight rise, and then stabilized at approximately 14 mm Hg without a significant variation in the IOP between 1 and 12 months. Traverso et al observed that the IOP was 15.3 ± 3.1 mm Hg (35% reduction) at 12 months after the Ex-PRESS miniature glaucoma shunt implantation.^[23]^ At 6 and 12 months after surgery, Konopińska et al^[24]^ observed that the IOP was 14.9 ± 3.6 and 17.1 ± 5.0 mm Hg, respectively. In our study, the IOP was 14.3 ± 3.9 mm Hg at 6 months, and was 14.0 ± 3.6 mm Hg at 12 months after surgery. In addition, the postoperative BCVA improved significantly at 12 months after surgery, and this improvement was the direct benefit of cataract extraction.

Similar to previous studies, the reduction of antiglaucoma medication used following implantation of the Ex-PRESS device was significant.^[7,23–25]^ In the present study, IOP-lowering treatment was not required for most of the patients postoperatively. Although the reintroduction of antiglaucoma medication occurred at the 2nd month after surgery, only 5 patients needed pharmacological treatment at their last visit: 3 were treated with 1 medication and 2 with 2 medications.

Nevertheless, our study revealed a better qualified success and complete success rate compared with previous studies,^[23,24,26]^ and there are probably 2 reasons for this outcome. For one hand, the time follow-up in this study was shorter than others; for the other hand, the pathogenesis of PACG was different from POAG. After combined surgery, the deepening of the anterior chamber, the widening of the drainage angle, and the improved access of aqueous to the trabecular meshwork may all contribute to the reduction of IOP and the number of antiglaucoma medications.

Advantages of the combined surgery are patient's convenience—a single operation, avoiding postoperative IOP spikes that can follow cataract surgery, especially dangerous in advanced optic neuropathy. However, it may be associated with more complications and additional surgery in postoperative period. In our study, a flat anterior chamber was the most common device-related complication observed after surgery. To prevent conjunctival erosion and to reduce early hypotony, the device was placed under a superficial scleral flap in our study, and no eyes developed erosion through the conjunctiva and exposure of the device. Compared with trabeculectomy, the Ex-PRESS procedure did not require a sclerectomy and peripheral iridectomy, it may decrease the early postoperative inflammation and hyphema.^[27]^ Bleb failure due to fibroblast proliferation is one of the main causes of failed filtration. Suture lysis and bleb needling were the most commonly reported additional procedures.^[27–29]^ In our study, there were 5 patients treated bleb needling with 5-FU injection, postoperatively.

In addition, Ex-PRESS implantation was better tolerated than trabeculectomy,^[5]^ and the device can offer a faster visual rehabilitation to operated patients, which is a primordial factor that should always be taken into consideration.^[30]^ Nevertheless, Ex-PRESS implantation is associated with higher surgical cost compared with trabeculectomy, and the higher cost is principally due to the cost of the device itself.^[31]^

Our study was limited by its retrospective nature and the number of patients enrolled (24) was relatively small. The recruited patients were not homogeneous in the sense that some had already received laser iridotomy before they were included in this study. In addition, in chronic PACG cases with severe PAS formation, cataract extraction alone may not be enough to re-open the angles and an insertion of Ex-PRESS in these cases may predispose to shallow anterior chamber or even Ex-PRESS-endothelial touch, so for these cases, trabeculectomy may be a better choice.

In summary, the Ex-PRESS implantation combined with phacoemulsification is safe and effective in reducing IOP and antiglaucoma medications in eyes with PACG and co-existing cataract. In some PACG cases not responding to maximum antiglaucoma therapy combined with senile cataract the implantation of Ex-PRESS miniature glaucoma shunt combined with cataract surgery could be the therapy of choice. Considering a short follow-up period in our study, a long-term follow-up period is needed in future research.
